# Toxicity and Residual Activity of Insecticides against *Diadegma insulare*, a Parasitoid of the Diamondback Moth

**DOI:** 10.3390/insects13060514

**Published:** 2022-05-31

**Authors:** Daniel Ramírez-Cerón, Esteban Rodríguez-Leyva, J. Refugio Lomeli-Flores, Lauro Soto-Rojas, Samuel Ramírez-Alarcón, Antonio Segura-Miranda

**Affiliations:** 1 Posgrado en Fitosanidad-Entomología y Acarología, Colegio de Postgraduados, Texcoco 56230, Estado de Mexico, Mexico; dceron150@gmail.com (D.R.-C.); esteban@colpos.mx (E.R.-L.); jrlomelif@hotmail.com (J.R.L.-F.); 2 Departamento de Parasitología Agrícola, Universidad Autónoma Chapingo, Chapingo, Texcoco 56230, Estado de Mexico, Mexico; ramirezsamuel@hotmail.com (S.R.-A.); antonioseguramiranda13@gmail.com (A.S.-M.)

**Keywords:** cruciferous crops, *Plutella xylostella*, natural control, integrated pest management

## Abstract

**Simple Summary:**

The diamondback moth is an insect pest that feeds on broccoli, cauliflower, cabbage, and other related plants. When its population increases, it can cause significant damage to those crops and economic losses to farmers. The fastest way to control this pest is by applying insecticides; however, the pest has become resistant to many of them. Therefore, it has been necessary to use higher doses of insecticides to decrease its population over time. At the same time, insecticides affect the pest’s natural enemies, which are essential to keeping pest populations low. *Diadegma insulare* is a tiny wasp that lays its eggs and develops inside the larvae of the diamondback moth, and kills it. This wasp is an important parasitoid of the pest and is found naturally in crop fields; it has also been introduced in some countries worldwide. In this research, we identified insecticides that are least toxic to *D. insulare*. If farmers have this information, they can choose products to kill the pest and reduce the impact of those products on natural field populations of *D. insulare*. By using the least toxic or residual insecticides, farmers will keep and increase the role of *D. insulare* as a natural enemy of the diamondback moth.

**Abstract:**

*Plutella xylostella* is the main pest of cruciferous crops worldwide. To reduce *P. xylostella* populations, better integration of natural control and chemical control (dominant tactic used) is needed. This work analyzed the compatibility of nine insecticides with the parasitoid *Diadegma insulare*, outlining them as complementary tools in an integrated pest management strategy. The acute toxicity of spinosad, imidacloprid, indoxacarb, flonicamid, naled, pyridalyl, emamectin benzoate, and spinetoram against the parasitoid was assessed. Residual activity (persistence) was also evaluated over time; the mortality of the parasitoid in contact with leaf tissue of plants treated with insecticides was analyzed. According to the International Organization of Biological Control, all nine insecticides were toxic to *D. insulare*; the lowest mortality was recorded with spirotetramat (64%) and pyridalyl (48%), while the rest of the insecticides caused 100% mortality at 72 h after application. In terms of persistence, by days 14, 16, 16, 17, 17, 21, and 22 after application, flonicamid, naled, spirotetramat, spinosad, piridalyl, imidacloprid, and indoxacarb caused mortality of less than 25%, respectively, so they were considered harmless (Category 1). Nonetheless, some insecticide toxicity and residual activity must be regarded within integrated pest management programs for conserving the role of *D. insulare* field populations.

## 1. Introduction

The diamondback moth, *Plutella xylostella* (L.) (Lepidoptera: Plutellidae), is the pest that causes the most significant economic losses in cruciferous crops worldwide, particularly of broccoli, cauliflower, cabbage, and Brussels sprouts [1,2,3]. Feeding larvae cause direct damage; a high larval population can reduce up to 70 or 80% production [4,5,6]. Nevertheless, indirect damage is considered more economically important than damage from feeding alone [7]. Indirect damage involves the presence of any larvae or their waste in the commercial product. Detection of a single larva in a head of broccoli inflorescence is considered unacceptable in commercial terms, and growers can be subject to restrictions if more than nine larvae are detected in 20 kg of marketable product from a full shipment of broccoli (13 tons) in the packing and freezing facilities before it is prepared for international trading [8,9].

International trade demands insect-free vegetables with specific aesthetic attributes which have led to production systems with minimal tolerance to pests and a high propensity for chemical control [10,11]. This situation cannot be better exemplified than by *P. xylostella* on cruciferous crops [3,12,13]. This pest has shown rapid response to selection pressure by insecticides of any toxicological group [14,15,16]. In the last two decades, some *P. xylostella* populations were reported resistant to some of the latest useful insecticides, among them diamides, spinosins, oxadiazines, and even to biological insecticides which contained *Bacillus thuringiensis* [17,18,19,20].

To avoid the indiscriminate use of insecticides and maintain low levels of *P. xylostella*, it is necessary to consider the importance of natural enemies within the Integrated Pest Management (IPM) approach [21,22]. There is much information that indicates acceptable levels of natural control of *P. xylostella* when natural enemies are allowed to develop in agricultural systems in the Mediterranean region, Africa, Asia, and North America [10,18,23]. One of this pest’s most promising natural enemies in Mexico and the United States of America is *Diadegma insulare* (Cresson) (Hymenoptera: Ichneumonidae). This solitary, provigenic, and specific endoparasitoid attacks third and fourth instar larvae of *P. xylostella*. Although it is not native to North America, it was introduced some decades ago and contributes to regulation of diamondback moth populations [4,5,24,25]. Parasitism of *P. xylostella* by *D. insulare* has reached 60 to 70% in some agroecosystems in California and Texas [18] and up to 45% in central Mexico [24,26].

The natural control of *P. xylostella* offered by *D. insulare* is conditioned by agronomic practices [4,27]. For example, in broccoli or cauliflower, *P. xylostella* has an economic threshold of 0.5 larvae per plant in the first 45–50 days after planting; this threshold drops to 0.2 larvae per plant (and even less) 50 days after planting to reduce risks that the larvae could move to the developing inflorescence head [13,28]. Avoiding or reducing the application of insecticides in the first 45–50 days can favor natural control of *P. xylostella* and other pests such as aphids, as several field studies have shown [9,13]. However, even following the best management practices for *P. xylostella*, some farmers must spray insecticides during the first 45 days after planting. The question is: Which insecticide can be considered less aggressive against the parasitoid *Diadegma insulare*?

There is no doubt that excessive use of insecticides depletes populations of natural enemies in crop fields [29,30]. Because *D. insulare* is one of the most common parasitoids of *P. xylostella* in cruciferous in North America, there is a high probability that spraying insecticides on those crops is impacting its natural populations [5,31,32,33]. Moreover, few studies have evaluated insecticide effects on *D. insulare* [5,34]. Therefore, the objective of this work was to characterize the toxicity and residual activity of insecticides on the parasitoid *D. insulare*.

## 2. Materials and Methods

### 2.1. Parasitoids

*Diadegma insulare* adults were obtained from the rearing farm La Huerta (Rancho Medio Kilo), located in Aguascalientes, Mexico. There, *D. insulare* was reproduced on *P. xylostella* fed on cabbage plants (*Brassica oleracea* var. Capitata). The parasitoids (<48 h old) were sent to the Colegio de Postgraduados, Estado de México, and kept in a rearing chamber at 23 ± 2 °C, 60 ± 5% RH in dark conditions for 24 h. Those conditions contributed to reduce mortality from mechanical damage. In the laboratory, adults were provided with a 3:1 water–fructose (Karo^®^ Corn Syrup, ACH Foods, Ciudad de Mexico, Mexico) solution *ad libitum*. All experiments were performed within 48 h of the arrival of the parasitoid adults to the laboratory. 

### 2.2. Laboratory Bioassay (Acute Toxicity)

The nine most recommended insecticides for controlling *P. xylostella* and other broccoli pests in Mexico and the USA were evaluated. Only registered formulations of different toxicological groups were included (Table 1). 

The medium concentration indicated on the label of the insecticides was evaluated; the products have a phytosanitary registration for the control of *P. xylostella*, aphids, or other pests (Table 1). Additionally, we used the surfactant Inex A^®^ (Cosmocel Iberoamerica, Monterrey, Mexico) at 1 mL/L of distilled water in each treatment. Two controls were included: distilled water (absolute control) and water plus the surfactant Inex A^®^. The volume of water per hectare indicated on the product label was used to prepare each treatment.

The bioassay used the methodology proposed in [35] with some modifications. Before applying the treatments, groups of 10 *D. insulare* adults were confined in a plastic Petri dish (2.5 cm in diameter) and anesthetized with CO_2_ for 20 s, for which a 1L hermetically sealed bag (Ziploc ^®^, SC Johnson, Chicago, WI, USA) was used. Then, the anesthetized insects were placed on absorbent paper on the bottom of a glass Petri dish (9.0 × 1.5 cm in diameter) for exposure to the insecticide inside a Potter tower (150 × 50 × 50 cm). The tower used a pneumatic solid cone spray nozzle (Cat. 1/4 J-SS + SU1A-SS, Spraying Systems, Wheaton, IL, USA), connected to a constant pressure air source. The system was calibrated to apply 2 mg cm^−2^ of insecticide by introducing 3 mL of solution at 20 PSI pressure. The experimental units were placed 120 cm away from the spray source. After the application of each treatment, the parasitoids were placed into clean plastic Petri dishes (4.0 cm in diameter) with holes and organza fabric for ventilation. A cotton swab with a 3:1 fructose–water solution was placed in each Petri dish. The parasitoids were kept at 23 ± 2 °C, 60 ± 10% RH in dark conditions; parasitoid mortality was evaluated 24, 48, and 72 h after application. Insects that could not move normally when stimulated with a small entomological brush (000 number) were considered dead. Each treatment had five repetitions, including the two control treatments (water alone and water–surfactant). Furthermore, the entire experiment was replicated twice using a different batch of parasitoids; a total of 100 parasitoids per treatment were evaluated.

### 2.3. Residual Activity (Persistence) of Insecticides to D. insulare

The experiment was conducted between August and October 2019 in an open field at the Colegio de Postgraduados, Campus Montecillo (19°27′51″ N 98°54′15″ W), at 22 ± 7 °C and 71–89% RH and an average dew point of 11.17. Seven commercial formulations of insecticides were evaluated. Emamectine benzoate and spinetoram products (Table 1) were not included due to the high toxicity observed in the laboratory bioassay and because the number of treatments had to be reduced for practical reasons. To evaluate the residual activity of the insecticides in semi-field conditions, 80 1.5-month-old broccoli plants (Imperial cultivar) were used. Plants were grown individually in plastic pots (6.2 L) containing tezontle (porous gravel of volcanic origin) + peat moss (Growing Mix^®^, Premier Tech Horticulture, Cromwell, MN, USA) (1:2). The plants were fertilized through an automated irrigation system, using the Ultrasol^®^ fertilizer (1 g/L water) (Soquimich Comercial, Santiago, Chile). 

To carry out the bioassay, we followed the methodology described in [36] with a few modifications. We used the middle concentration indicated on the label of each of the insecticides evaluated (Table 1); additionally, the Inex A^®^ surfactant was used at 1 mL/L of water to prepare each treatment. Individual 1.5-month-old broccoli plants were sprayed to runoff with the corresponding insecticide concentration using a manual backpack sprayer (Swissmex 425^®^, Jalisco, Mexico; 15 L capacity at 40 PSI) fitted with a full cone nozzle. When spraying the plants, the upper and underside of the leaves were uniformly covered. All applications were made on the same day. The sprayer was thoroughly flushed to remove residue; control plants were treated with tap water plus the surfactant only. There were 10 replicate plants for each treatment, randomly distributed. A distinctive label was placed for the plants of each treatment; this guaranteed the correct selection of leaves for the evaluation of the residual activity. All plants remained in direct sunlight, and dissipation similar to that which occurs in field conditions was induced. At specific times when there was a high probability of rain, the plants were protected with a plastic cover.

Broccoli leaves were taken randomly within each treatment 24 h after spraying and then every 72 h up to Day 1 (8 sampling dates); the sampled leaves were transferred to the laboratory, where several leaf discs (6 cm in diameter) were cut from each treatment. Leaf discs were placed in a plastic Petri dish (6 cm in diameter and 5 cm deep) on a 2 mm layer of water agar (2.5% *w*/*v*); each Petri dish had two of these leaf discs; a leaf disc was placed on the agar in the bottom and another on the agar in the top of the dish. Additionally, each Petri dish had 4 lateral holes (1 cm in diameter), 3 of which were covered with organza fabric to promote ventilation, and 10 *D. insulare* adults (<48 h of age) were introduced through the other hole (0.5 cm in diameter), which was then sealed with water-saturated cotton. In the organza fabric of each ventilation hole, thin lines of honey were offered to the parasitoid adults every day. The Petri dishes were randomly arranged inside a rearing chamber at 23 ± 2 °C, 60 ± 10% RH in dark conditions. Mortality was assessed 24 h after exposing the parasitoids to treated leaf discs. To determine if a parasitoid remained alive, it was stimulated with a fine camel-hair brush (000); if no reaction was shown, it was considered dead. In each evaluation, 5 Petri dishes were examined per treatment (each dish with 10 adult parasitoids); 400 parasitoids were used per treatment for the 8 evaluation dates.

### 2.4. Analysis of Data

Data from the laboratory bioassay were subjected to a one-way analysis of variance (ANOVA), and arcsine transformation was applied to satisfy the assumptions of the parametric analysis. In cases where differences were detected, a multiple separation of means test was performed (Tukey, α = 0.05). Parasitoid mortality was classified using the 4 laboratory bioassay toxicity categories proposed by the International Organization for Biological Control (IOBC): Category 1 (harmless, <30% mortality), Category 2 (slightly harmful, 30–79% mortality), Category 3 (moderately harmful, 80–99% mortality), and Category 4 (highly harmful, >99% mortality) [37]. For the residual activity assay, mortality, expressed as a proportion of all the insects evaluated, was analyzed by fitting a logistic regression model to obtain a dissipation curve. The analyses were carried out with the programming language R version 4.0 [38]. The adjusted logistic regression model was used to describe the toxicity and persistence of each insecticide according to the IOBC classification categories to describe pesticide activity against natural enemies. The classification is: Category 1 (harmless, <25% mortality), Category 2 (mildly harmful, 25–50% mortality), Category 3 (moderately harmful, 50–75% mortality), and Category 4 (harmful, >75% mortality). Persistence is classified as Category 1 (short-lived, non-persistent, harmless after <5 days), Category 2 (slightly persistent, harmless after 5–15 d), Category 3 (moderately persistent, harmless after 16–30 d), and Category 4 (persistent, becomes harmless only after >30 d). Harmless refers to less than 30% mortality in laboratory conditions and 25% mortality in semi-field, field, and greenhouse conditions [39].

## 3. Results

### 3.1. Laboratory Bioassay (Acute Toxicity)

Diadegma insulare was susceptible to all insecticides tested, but there were significant differences in susceptibility among treatments at 24 h (F_10,99_ = 653.54; *p* ≤ 0.0001). Moreover, cumulative mortality increased for some insecticides at 48 h (F_10,99_ = 3178.81, *p* ≤ 0.0001) and 72 h (F_10,99_ = 953.16; *p* ≤ 0.0001). At 72 h after exposure, the absolute control (distilled water) did not exceed 3% mortality, but Inex, A^®^ + distilled water (27% mortality) and the insecticides pyridalyl (48%) and spirotetramat (64%) increased more than double their percentage of mortality compared to 24 h (Table 2). According to the IOBC laboratory categories, only water (absolute control) and Inex A^®^ + distilled water were classified as harmless for D. insulare. The spirotetramat and pyridalyl treatments were slightly harmful (Category 2), and all other treatments were highly harmful (Category 4) (Table 2).

### 3.2. Residual Activity (Persistence) of Insecticides to D. insulare

**Figure 1:**
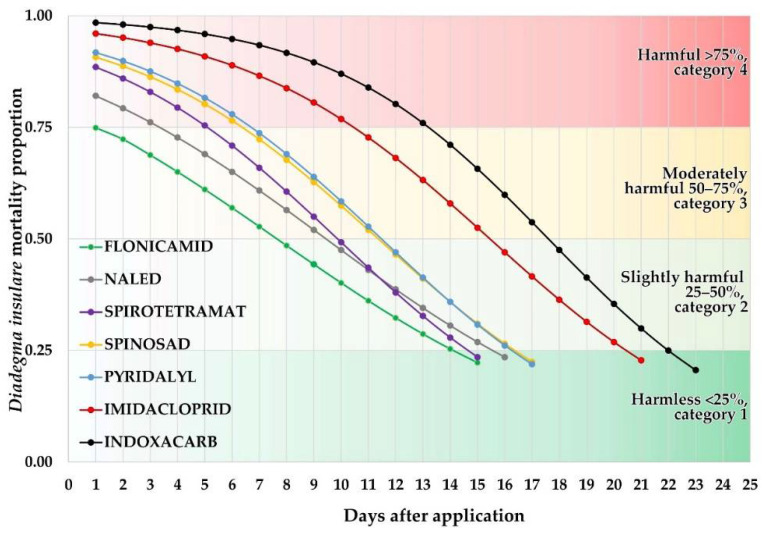
Toxicity and persistence of seven insecticides against adult *Diadegma insulare* on broccoli plants grown in an open field; lines adjusted after Abbott transformation [40].

The highest mortality rates were recorded in the first evaluation (24 h after application); this variable ranged between 75% (flonicamid) and 96% (imidacloprid) (Figure 1). The effect of the treatments dissipated over time, so their toxicity gradually decreased, although the rate of reduction was different for each treatment; six insecticides were toxic and harmful (Category 4, >75% mortality) at the 24-h evaluation, but imidacloprid and indoxacarb were the most toxic to parasitoids, and their effect persisted longer than the other insecticides, all of which caused <25% mortality within 15–17 days (Figure 1y2).

**Figure 2:**
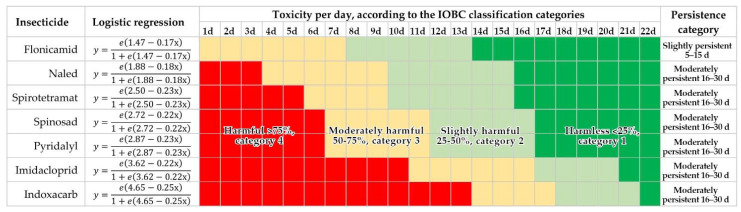
Toxicity and persistence of insecticides on *Diadegma insulare* using the IOBC classification.

Logistic regression modeled the effect of insecticides and their dissipation over time; this helped us to classify insecticides by their toxicity and persistence (Figure 1y2). The least toxic insecticide in the 24 h evaluation was flonicamid (Category 2, moderately harmful). It also had the shortest period of residual activity against the parasitoid and reached Category 1 (harmless) 14 days after treatment. Therefore, this product was classified as slightly persistent (Category 2). Pyridalyl, spirotetramat, spinosad, and naled were harmful (Category 4, >75% mortality) at 24 h and remained harmful 15 to 17 d before reaching the harmless Category 1. Imidacloprid and indoxacarb had a longer residual effect for approximately 7 d before they became harmless (Category 1), compared to the first four insecticides. However, all were classified as moderately persistent (Category 3) according to the IOBC classification (Figure 2).

**Figure 3:**
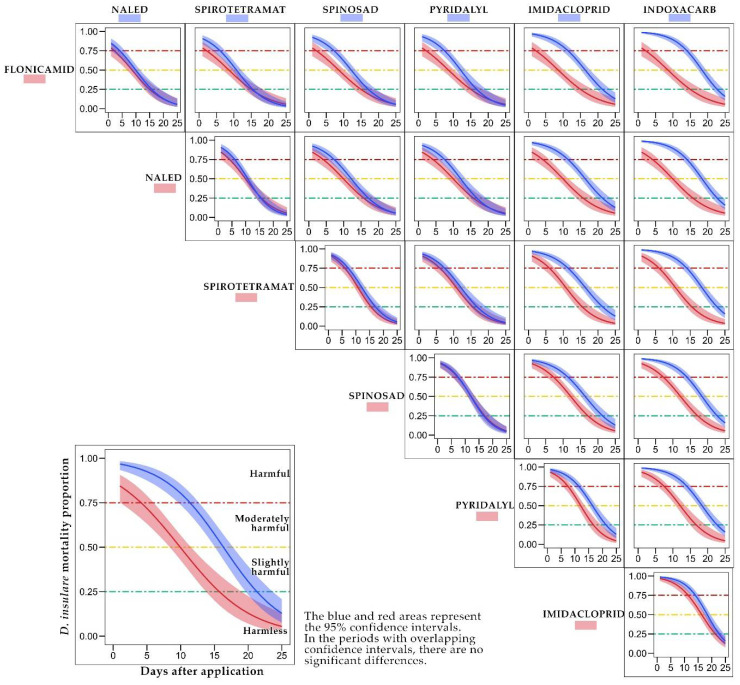
Comparison of the adjusted models using the 95% confidence intervals.

Imidacloprid and indoxacarb did not have significant differences in the models that describe the rate of dissipation of their toxicity over time. However, both insecticides acquired the category of harmless (<25% toxicity) in a significantly more extended period than the treatments flonicamid, naled, spirotetramat, spinosad, and pyridalyl (Figure 3).

## 4. Discussion

*Diadegma insulare* was affected by nine insecticides that are recommended for control of *P. xylostella* and other primary pests in broccoli. Although acute toxicity varied depending on the type of insecticide, seven insecticides caused 100% mortality 24 h after spraying. In contrast, pyridalyl and spirotetramat caused 48 and 64% mortality 72 h after spraying, respectively. Acute mortality of *D. insulare* by spraying was consistent with prior reports that have shown that most insecticides used on broccoli are highly toxic against hymenoptera and other beneficial arthropods [5,34,41,42]. Moreover, less than 65% mortality by direct aspersion of pyridalyl and spirotetramat on other hymenopterans and predators such as *Orius stringicollis*, *Harmonia axyridis*, and *Bombus terrestris* has been registered [43,44,45,46]. 

Evidence from our bioassay showed that *D. insulare*, like most parasitoids, is very susceptible to aspersion of almost any of the insecticides recommended for control of broccoli pests. Therefore, the rational use of these products will require assessment of residuality to favor the regulation of pest populations [47,48]. 

The residual activity of chemical products was evaluated on contaminated leaves, and the most toxic insecticides were indoxacarb and imidacloprid, contact and systemic insecticides which are known to produce paralysis because they attack the nervous system [30,49] and are also persistent in the environment [50]. In our assays, both products registered the longest residuality against *D. insulare* of all the insecticides tested. This likely was because *D. insulare* was in contact with residues on the contaminated leaf and showed self-grooming and cleaning constantly; *D. insulare* walked and stayed constantly on the leaf disc, and some insecticide molecules may be absorbed through the integument or the mouth because of the grooming behavior [51]. High toxicity of imidacloprid and indoxacarb persisted for 21 and 22 d, respectively, in the field (Figure 2). 

In contrast, chemical products such as flonicamid, naled, spirotetramat, pyridalyl, and spinosad, with different modes of action, did not persist more than 16–17 d. This likely was because of the mode of action of the insecticides and their susceptibility to the environmental conditions on broccoli leaves. Because many of these insecticides might have sublethal effects on parasitoids [29,52], it is important to mention that acute toxicity, which was the only determined here, would not be sufficient to consider that they are completely safe, and more evaluations are necessary to determine their full effect on *D. insulare* adults. However, considering that these products caused lower mortality of *D. insulare* 15–17 d after field application (<25%), which is considered safe according to the IOBC classification, these products might be considered more compatible with the parasitoid. If insecticide application against pests is needed in broccoli, these products might allow natural populations of *D. insulare* to reestablish in the field faster than products such as imidacloprid or indoxacarb.

We did not find studies on residual insecticide activity on *D. insulare*, but we searched the literature with results on predators and other parasitoids to comment on the residual activity of certain products. Some insecticides such as flonicamid and spirotetramat, which penetrate systemically through the leaf surface, could be less able to cause mortality by contact a few days after application. For example, in a previous study [53], using spirotetramat in lab tests on *Tamarixia radiata* (Waterston) (Hymenoptera: Eulophidae) was considered safe. In that study, they sprayed 179 mg a.i. per liter on pink grapefruit leaves, and contaminated leaves caused 26.1% mortality of the parasitoid after 24 h, and just 2.9% mortality after 22 d. In another parasitoid species of the same genus (*Tamarixia triozae* Burks), sprayed tomato leaves at the highest recommended concentration of spirotetramat (480 mg a.i. per liter) registered 42% mortality after 24 h of the insecticide application, and it was reduced to 22% after 7 d; leaf waxiness may have played a role in residual activity of the insecticide [36].

Despite some reports indicating that spinosad has low toxicity for some insect natural enemies [52,54], other studies reported the opposite [12,36,55]. Our study established that spinosad persists nearly 17 d (25% mortality) on broccoli leaves in the field. Like any insecticide applied to field crops, spinosad is exposed to daylight and rain, and the degradation because of the sunlight is one of the important factors for it [41,56]. Moreover, Ref. [54] reported that depending on the sunlight received, the residual activity of spinosad had a range of 1.6 to 16 d. The residual activity of spinosad in this study was likely due to radiation, temperature, morning dew, leaf waxiness, and the plant canopy (rain was prevented in our experimental essays). These conditions are typical on the high plateau in Central Mexico and in some broccoli production regions in Mexico and other countries. The persistence of pyridalyl (17 d) and naled (16 d) was very similar, but we did not have an explanation of how these contact and contact and ingestion products were able to last that long. However, toxic molecules of both products were able to remain on broccoli leaves under the environmental conditions already described. 

The difference in persistence of insecticides against *D. insulare* can be associated to different factors. For example, susceptibility of the wasp, leaf waxiness on broccoli, absorption of the compound after spraying, and the environmental conditions where and when the assay was developed. We established that flonicamid can be categorized as slightly persistent (Category 2), and the other six insecticides (naled, spirotetramat, spinosad, pyridalyl, imidacloprid, and indoxacarb) can be classified as moderately persistent (Category 3, 16–30 d). Nevertheless, the persistence of imidacloprid and indoxacarb persisted longer numerically (21 and 22 d) than the first four insecticides (16–17 d). This should be considered to promote the reestablishment of natural *D. insulare* populations in the field where its role as a regulator of *P. xylostella* populations has been demonstrated. 

## 5. Conclusions

Considering the role of natural control on *P. xylostella* in agroecosystems of broccoli in the world and the parasitism of *D. insulare* as one of the biotic factors, we conclude that insecticides that showed less residual activity can potentially be used in IPM programs. We also would like to emphasize that this study only evaluated the acute toxicity of insecticides, and sublethal effects on *D. insulare* must be evaluated to understand their impact on this natural enemy fully.

## Figures and Tables

**Table 1:** Insecticides and doses evaluated in acute and residual toxicity tests in adults of the parasitoid *Diadegma insulare*.

Active Ingredient (a.i.)	Commercial Name	Concentration (mg a.i. L^−1^)	Recommended Dose	Toxicological Group	Mode of Action
Emamectin Benzoate	Proclaim^®^ 05 SG	5.00	0.30–0.40 kg/ha	Avermectin	Glutamate-gated chloride channel allosteric modulators. Acts on the nervous system and muscle system.
Spinosad	Spintor^®^ 12 SC	44.20	0.10 L/ha	Spinosins	Nicotinic acetylcholine receptor allosteric modulators; acts on the central nervous system.
Spirotetramat	Movento^®^ 150 OD	15.30	0.3–0.4 L/ha	Tetronic acid	Inhibitors of acetyl CoA carboxylase; inhibit lipid biosynthesis and growth regulator.
Imidacloprid	Confidor^®^ 350 SC	35.00	0.15–0.30 L/ha	Neonicotinoid	Nicotinic acetylcholine receptor competitive modulators; acts on the nervous system.
Spinetoram	Palgus^®^	5.87	0.25–0.30 L/ha	Spinosins	Nicotinic acetylcholine receptor allosteric modulators; acts on the central nervous system.
Indoxacarb	Avaunt^®^ 150 EC	15.84	0.30–0.50 L/ha	Oxadiacines	Voltage-dependent sodium channel blockers. Acts on the nervous system.
Flonicamid	Beleaf^®^	50.00	0.10–0.25 kg/ha	Pirydinecarboxiamides	Chordotonal organ modulators undefined target site; acts on the nervous system.
Naled	Dibrom^®^ 8	66.5	0.750–1.25 L/ha	Organophosphates	Acetylcholinesterase inhibitors; acts on the nervous system.
Pyridalyl	Pleo^®^ 50 EC	44.9	0.20–0.35 L/ha	Derivatives of dihaloprene	Compounds of an unknown or uncertain mode of action.

**Table 2:** Diadegma insulare mortality 24, 48, and 72 h after insecticide application.

Treatment	Cumulative Mortality (%±EE) ^1^			IOBC Toxicity Categories ^2^ at 72 h
	24 h	48 h	72 h	
Indoxacarb	100 ± 0.0 a	100 ± 0.0 a	100 ± 0.0 a	Highly harmful (Category 4, >99% mortality)
Emamectine benzoate	100 ± 0.0 a	100 ± 0.0 a	100 ± 0.0 a	
Imidacloprid	100 ± 0.0 a	100 ± 0.0 a	100 ± 0.0 a	
Naled	100 ± 0.0 a	100 ± 0.0 a	100 ± 0.0 a	
Spinoteram	100 ± 0.0 a	100 ± 0.0 a	100 ± 0.0 a	
Spinosad	100 ± 0.0 a	100 ± 0.0 a	100 ± 0.0 a	
Flonicamid	100 ± 0.0 a	100 ± 0.0 a	100 ± 0.0 a	
Spirotetramat	26.0 ± 0.6 b	64.0 ± 0.2 b	64.0 ± 0.2 b	Slightly harmful (Category 2, 30–79% mortality)
Pyridalyl	18.0 ± 0.3 b	38.0 ± 0.2 c	48.0 ± 0.5 c	
Water + InexA	10.0 ± 0.0 c	27.0 ± 0.2 d	27.0 ± 0.2 d	Harmless (Category 1, <30% mortality
Control	1.11 ± 0.0 c	1.11 ± 0.0 e	2.2 ± 0.0 e	

^1^ Means with the same letter in a column are not statistically different (*p* ≤ 0.05); ^2^ Categories for laboratory bioassays according to the International Organization for Biological Control. (IOBC) [39].

## Data Availability

The data presented in this study are available on request from the corresponding author. The data are not publicly available due to the authors would like to know how the requested data will be used.
